# Parliament and the Profession

**Published:** 1917-04-28

**Authors:** 


### Parliament and the Profession.

### Deaths at Kinmel Park Camp.

Mr. Macpherson gave Mr. Haydn Jones details of the deaths which occurred at Kinmel Park Camp in February and March. Two officers and thirty-two men had died. The causes included thirteen from pneumonia and four from cerebro-spinal meningitis. Two of the deaths were the result of accidents.

### Tuberculosis in County Meath.

Mr. Duke, replying to a question of Mr. P. White, stated that as the Local Government Board of Ireland, in agreement with the Irish Insurance Committee, had advised that the treatment of tuberculosis in County Meath was not satisfactory, the Treasury had decided not to sanction the usual grant.

### Disabled Soldiers' Artificial Limbs.

Major Chapple, in a question to the Minister of Pensions, called attention to the high cost of artificial limbs under present conditions of supply. Mr. Barnes stated that the main consideration the Government had in view was not the cheapness of the limb but its efficiency. A committee of experts exists for the purpose of advising as to the most suitable types of limbs, and as to the prices which should be paid for them. If they advise that prices are excessive, the question of a Government factory will be considered.

### Disabled Soldiers and Crutches.

Sir G. Toulmin, in a question to the Under-Secretary of State for War, called attention to a statement made by a French doctor, quoted by Sir Henry Norman, M.P., in his report on the treatment, etc., of disabled soldiers, as to the effect of the continued use of crutches, and was informed by Mr. Macpherson that the old pattern T-crutches have now been discarded and replaced by a new pattern, with adjustable horizontal handles, which admit of the weight of the body being borne by the hands, and guard against possible ill-effects of the old pattern.

